# Water-enhanced antegrade MR pyelography in pregnancy: a novel radiation-free approach

**DOI:** 10.1186/s41747-017-0019-0

**Published:** 2017-10-27

**Authors:** Grainne N. Allen, Bryan W. Buckley, Daniel M. Conroy, Dara J. Lundon, Padraic MacMathuna, Kiaran O’Malley, Leo P. Lawler, Carole A. Ridge

**Affiliations:** 1 Mater Misericordiae University Hospital, Dublin, Ireland; 2 University College Dublin, Dublin, Ireland; 3 Belfast City Hospital, Belfast, UK

**Keywords:** Magnetic resonance imaging, Percutaneous nephrostomy, Antegrade MR pyelography, Pregnancy, Urologic diseases

## Abstract

Our aim was to determine if water-enhanced antegrade magnetic resonance (MR) pyelography can be an alternative to conventional antegrade pyelography in pregnant patients who require percutaneous nephrostomy placement for urosepsis and/or obstructive uropathy. The pregnant patient was placed supine in a 1.5-T MRI scanner seven days after percutaneous nephrostomy placement using ultrasound. Serial axial and coronal T2-weighted echo-planar fast spin-echo sequences were performed before and after injection of the catheter. The right nephrostomy catheter hub was sterilised using chlorhexidine. Sixty millilitres of sterile water were slowly injected. No Gd-based contrast agent was utilised due to safety concerns for the foetus. MR antegrade pyelography demonstrated the level of ureteric obstruction and the absence of renal calculi using sterile water as a contrast medium injected through a percutaneous nephrostomy followed by T2-weighted imaging. Air bubbles in the injected solution were differentiated from calculi due to their mobility on serial scans and their anti-dependent position. Water-enhanced antegrade MR pyelography was a safe and effective method of imaging the pregnant patient. It served as an alternative to conventional antegrade pyelography and minimised potential risks to the foetus.

## Key Points

- The European Society of Urogenital Radiology cautions the use of Gd-based contrast agents in pregnancy.
- Nephrostomy is an invasive technique which is indicated in pregnant women with obstructive uropathy complicated by urosepsis or renal failure.
- Water-enhanced antegrade MR pyelography through percutaneous nephrostomy is an alternative to fluoroscopic pyelography.
- Air bubbles are differentiated from calculi by their mobility and anti-dependent position.

## Introduction

Urosepsis due to obstructive uropathy is a rare complication of pregnancy [1], occasionally requiring placement of a percutaneous nephrostomy. Fluoroscopic antegrade pyelography may be indicated to determine the level and cause obstruction. Although typical fetal doses from diagnostic radiology studies are usually low, it is important to keep fetal radiation doses as low as possible [2, 3]. Antegrade pyelography, however, requires the use of iodinated contrast medium and ionising radiation exposure. Magnetic resonance (MR) imaging can circumvent the latter challenge [4–8] but the potential effects of Gd-based contrast agent on the foetus have not been determined, with one animal study suggesting teratogenicity [9]. The European Society of Urogenital Radiology (ESUR) advises caution when using Gd-chelates in pregnant women [10]. This report highlights a potential alternative to the use of ionising radiation and conventional contrast agents in the pregnant woman with obstructive uropathy.

## Case description

A pregnant 31-year-old woman at 22 weeks’ gestation presented with a 48-h history of right renal angle pain, nausea, decreased appetite and diarrhoea. The patient was afebrile. The blood pressure was 90/59 and pulse was 87 beats per minute. Foetal movements were normal. Laboratory values included a neutrophilia of 9 × 10^9^/l, a serum C-reactive protein of 66 mg/l and creatinine of 66 μmol/l. Urinary microscopy demonstrated white cells of > 1000/μl with positive urinary cultures for *Escherichia coli*. The patient consented to the clinically indicated imaging tests and procedures performed including a detailed discussion of the associated foetal risks with each intervention and consent to the use of anonymised images for research purposes.

Ultrasound was performed using a curvilinear 3–5-MHz abdominal ultrasound probe (Toshiba, Tokyo, Japan). This demonstrated moderate right hydronephrosis and a percutaneous nephrostomy was inserted under ultrasound guidance to treat suspected urosepsis confirmed by the clinical observation of pyuria in the drainage bag after nephrostomy placement.

A day later, MR imaging of the abdomen was performed to determine the level and cause of obstruction. The examination confirmed decompression of the right renal collecting system but the level and cause of obstruction could not be determined due to non-distension of the right renal pelvis and ureter. The patient’s symptoms had resolved.

Seven days after nephrostomy placement, the referring urologic surgeon considered the removal of the nephrostomy after a successful clamping trial of 24 h but wished to determine if the obstruction was due to renal calculi or physiological compression by the gravid uterus. In the light of the unrevealing MR imaging, antegrade pyelography appeared to be indicated. However, a solution that minimised the patient and foetal exposure to ionising radiation was sought.

Gd-enhanced urography was considered, but following a literature search, which confirmed that the potential effects of Gd-chelate adminisitration on the foetus had not been determined yet, and one animal study suggesting teratogenicity [9], the decision was taken to perform antegrade MR pyelography using sterile water and T2-weighted imaging.

**Fig. 1:**
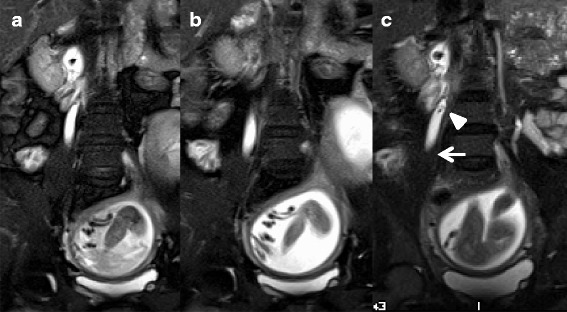
Coronal HASTE images of the abdomen and pelvis in a 31-year-old pregnant woman at 22 weeks’ gestation. Serial images demonstrate blooming artefact due to a nephrostomy in the right renal pelvis (**a**, **c**), and gradual tapering of the right ureter towards the level of the gravid uterus at the pelvic brim (**b**, **c**, *arrow*). Transient filling defects in the mid ureter (**c**, *arrowhead*) represent gas bubbles

**Fig. 2:**
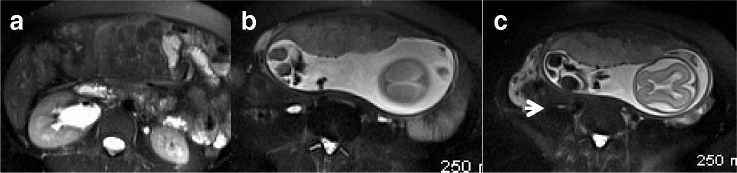
Axial HASTE images of the abdomen and pelvis in a 31-year-old pregnant woman at 22 weeks’ gestation. Serial images demonstrate anti-dependent air bubbles in the right renal pelvis (**a**) and gradual tapering of the right ureter towards the level of the gravid uterus at the pelvic brim (**b**, **c**, *arrow*)

The patient was placed supine in a 1.5-T Magnetom Symphony scanner (Siemens, Erlangen, Germany). A T2-weighted half Fourier acquisition single-shot turbo spin-echo (HASTE) coronal sequence was performed as a control prior to injection with a standard body coil (excitation time 66 ms, echo train length 128, flip angle 180°, number of excitations 1, matrix 240 × 256, slice thickness 4 mm, TA (acquisition time) 13 s). The right nephrostomy catheter hub was sterilised using chlorhexidine and 60 mL of sterile water were slowly injected. Serial coronal and axial HASTE sequences were performed and reviewed immediately by the radiologist to confirm adequacy. Antegrade MR pyelography confirmed gradual tapering of the right lower ureter just above the level of the gravid uterus (Fig. 1). No calculus was demonstrated at this level. Non-dependent filling defects in the right renal pelvis and upper ureter were confirmed to be transient on serial scanning, consistent with small air bubbles due to hand injection (Fig. 2). Antegrade MR pyelography confirmed the cause of urosepsis to be a physiologic obstruction due to the gravid uterus. The nephrostomy was clamped again for a further 24 h; the patient remained asymptomatic thus clinically excluding ongoing obstruction. The nephrostomy was therefore removed and the patient remained well at 12-month clinical follow-up.

## Discussion

This report outlines a novel use of MR imaging in pregnancy complicated by urosepsis requiring nephrostomy placement. Antegrade MR pyelography successfully determined the cause of obstruction in this pregnant woman without the use of Gd-chelates or ionising radiation. The utilisation of T2-weighted imaging alone eliminated the need for iodinated or Gd-based contrast media and facilitated removal of the percutaneous nephrostomy once urosepsis had resolved and ongoing obstruction had been clinically excluded by a clamping trial. In fact, the safety of Gd-based contrast media in pregnancy and their reported potential for teratogenicity were concerns in this case. A literature review prior to antegrade MR pyelography found no evidence of mutagenic or teratogenic effects to the foetus after administration of Gd-chelates [11]. However, an animal study reported a slightly higher incidence of early intrauterine deaths in rabbit foetuses whose mothers had been administered with high doses of intravenous gadobenate dimeglumine [9]. A second animal study reported that repeated doses of Gd-based contrast agent in healthy rats resulted in cerebellar deposition of Gd in the cerebellum [12]. The ESUR guidelines advise that only small doses of the most stable Gd-based contrast agents should be given to pregnant women and that the use of these agents should be fully discussed with the patient [10, 13]. In this case, water-enhanced MR pyelography presented an attractive option to minimise maternal anxiety and potential side effects as well as introduce another novel application for MR imaging.
